# Supplementary material for: Dynamically expressed single ELAV/Hu orthologue elavl2 of bees is required for learning and memory
Source: Commun Biol. 2021 Oct 28;4:1234. doi: 10.1038/s42003-021-02763-1 (PMC8553928; doi:10.1038/s42003-021-02763-1)
Supplement: Supplementary file 2 — Supplementary Information [file 42003_2021_2763_MOESM2_ESM.pdf]

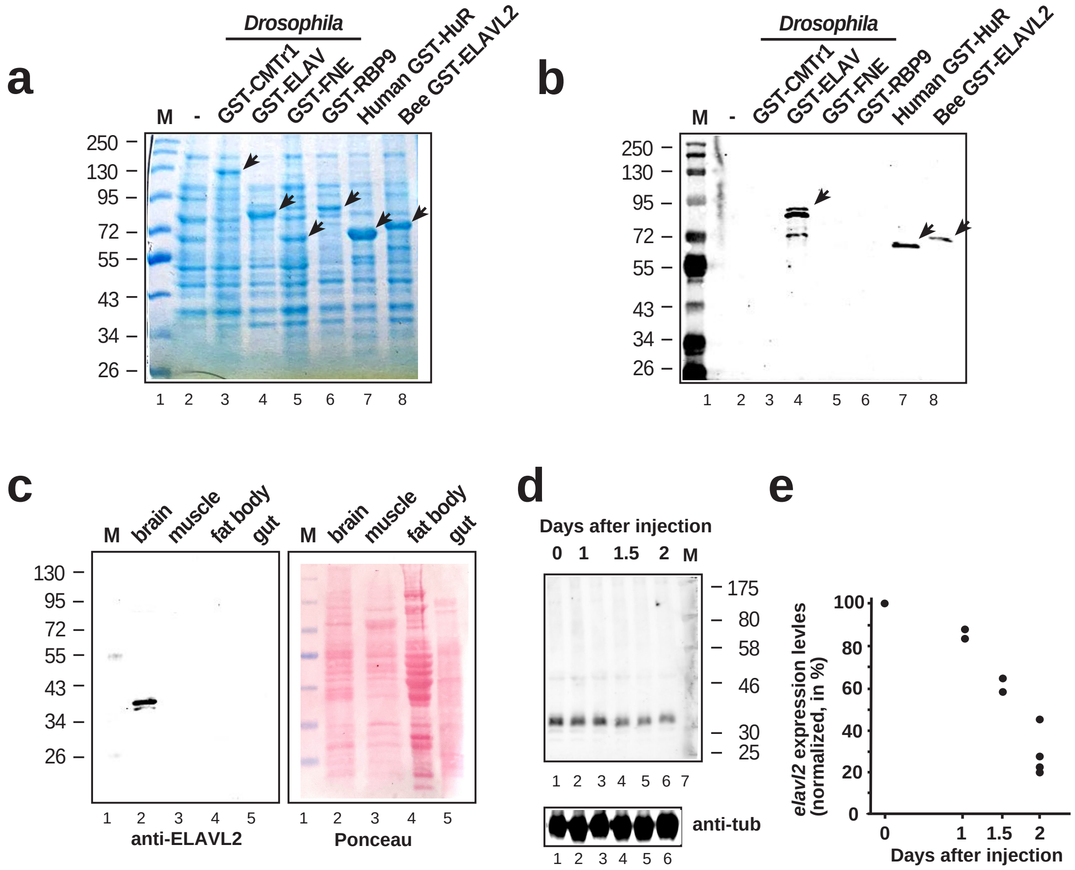

Supplementary Fig. 1 Specificity of a rat polyclonal anti-serum raised against *Drosophila* ELAV. a, b A polyclonal anti-serum raised against *Drosophila* ELAV cross-reacts with *Drosophila* ELAV, bee ELAVL2 and human HuR. Recombinant GST-fusion proteins for *Drosophila* ELAV (78 kDa), FNE (65 kDa) and RBP9 (74 kDa), for *Apis* ELAVL2 (68 kDa), for human HuR (66 kDa) and for control *Drosophila* cap methyltransferase CMTr1 (126 kDa) expressed in *E. coli* were analyzed on a 10 % Coomassie stained SDS-gel (**a**) and a 1:100 dilution was probed with a polyclonal anti-serum raised against *Drosophila* ELAV on a Western blot (**b**). Molecular weight markers are shown on the left. **c** Bee ELAV is expressed in brains, but not muscle tissue, fat body or gut detected on Western-blots probed with a rabbit polyclonal anti-ELAV anti-serum (left) and protein content stained with Ponceau (right). Molecular weight markers are shown on the left. **d, e** Time-course of ELAVL2 reduction by RNAi different times after injection into the bee brain shown on a representative Western probed with polyclonal anti-ELAV anti-serum (top) or tubulin (bottom), and quantification (**e**).

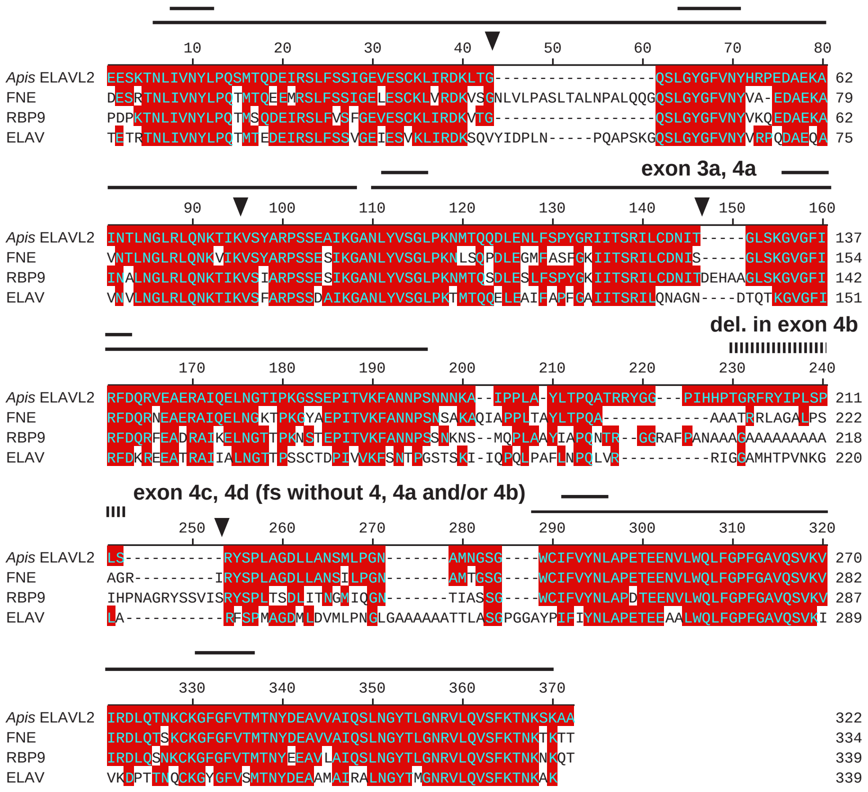

Supplementary Fig. 2 Sequence comparison of ELAV family proteins of bees and *Drosophila*. Alignment of single *Apis mellifera* ELAVL2 (XM006571184) with the three *Drosophila melanogaster* orthologues ELAV (AAF45517), FNE (AAF48215) and RBP9 (AAF51177). Long and short lines on top indicate the three RRMs and RNP motives, respectively. Black triangles depict intron positions. Alternative exons 3a, 4a, 4c and 4d are indicated at relevant intron positions. The hatched line indicates the sequence deleted in exon 4b by alternative splicing. Note that skipping of exons 4, 4a or 4b will result in a truncated protein due to a frameshift (fs).

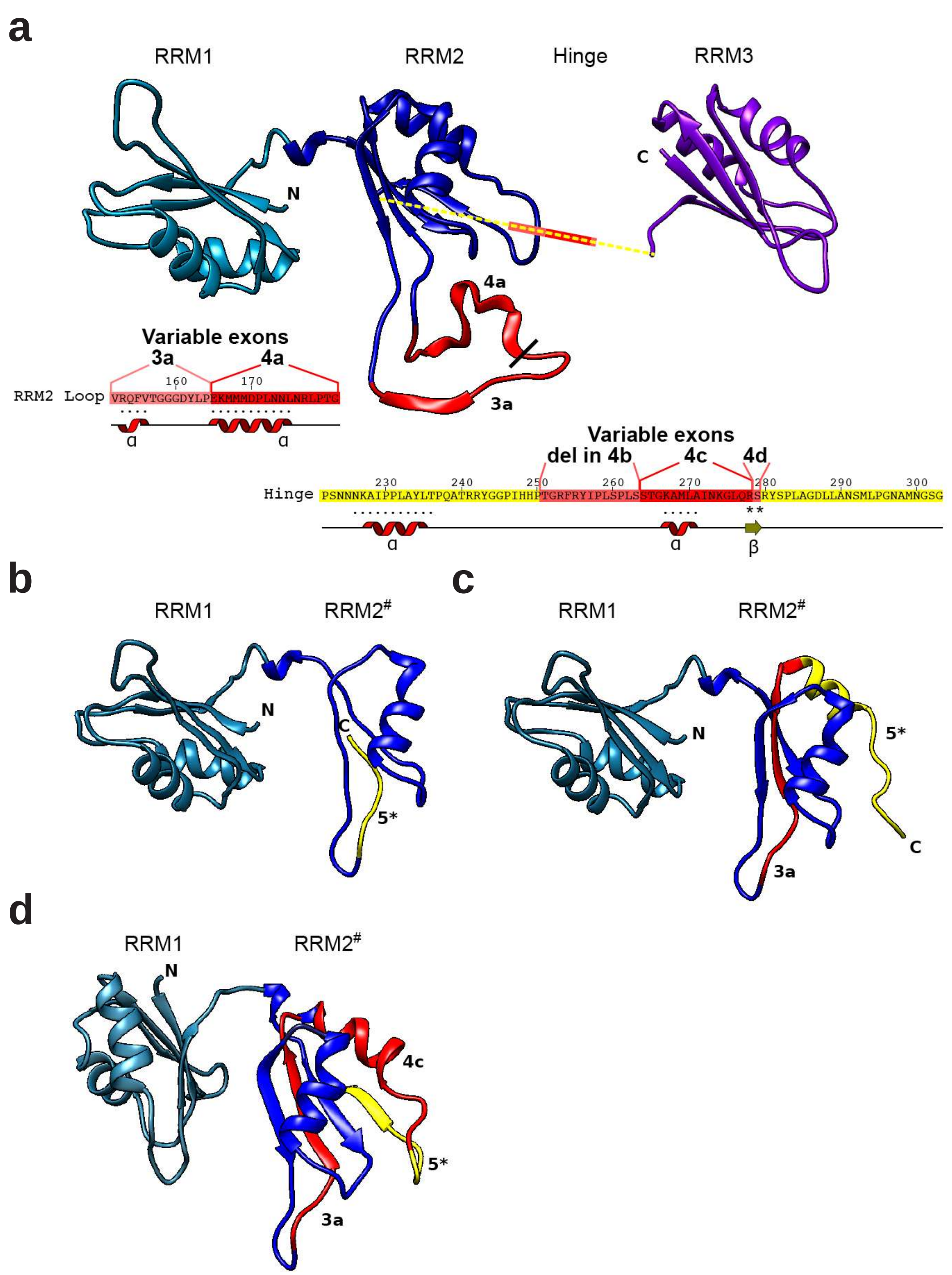

Supplementary Fig. 3 Structural models of bee ELAVL2 depicting the position of alternative exons. a Full length ELAVL2. The hinge region is indicated by a dashed line with the position of alternative exons 4b-d in red. Note that alternative exons are in flexible linker regions in RRM2 (exons 3a and 4a) and the hinge region (exons 4b, 4c and 4d). Structure predictions for alternative exons 3a and 4a are shown on the left and for the hinge region to the right with the sequence on top and structure features at the bottom. Dots and asterisks in between indicate amino acid in structured parts. N- and C-termini are indicated by "N" and "C", respectively. **b** Truncated ELAVL2 resulting from skipping of exon 4. The part in exon 5 is indicated in yellow and the asterisk denotes a frameshift resulting in a different sequence than in full length ELAVL2. The partial nature of RRM2 is denoted by a hashtag (#). **c** Truncated ELAVL2 resulting from inclusion of exon 3a and skipping of exon 4. Note that exon 3a adds an additional beta-sheet and the frameshifted exon 5* (yellow) adds an alpha helix to truncated RRM2# potentially increasing RNA binding. **d** Truncated ELAVL2 resulting from inclusion of exons 3a and 4c, and skipping of exon 4. Note that exon 4c adds a second alpha-helix and the frameshifted exon 5* (yellow) adds a beta-sheet to truncated RRM2#.

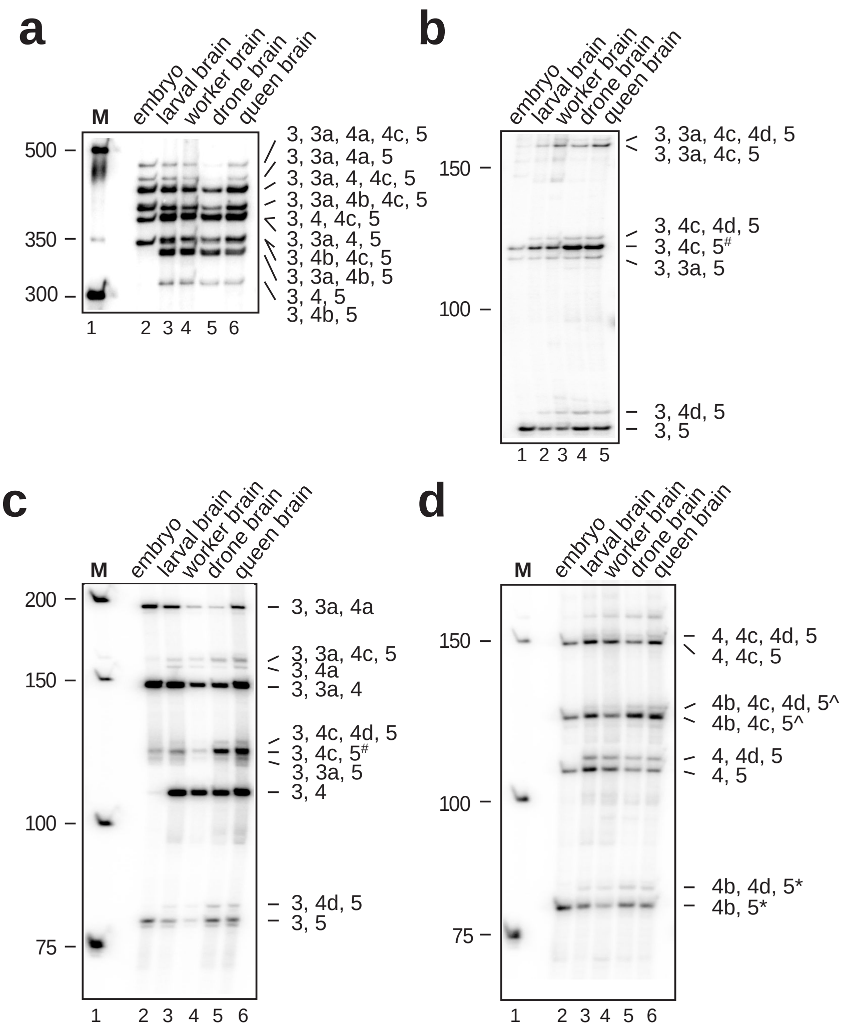

Supplementary Fig. 4 Representative gels from the developmental analysis of ELAVL2 alternative splicing quantified in Fig. 3g-i. a, b Top and bottom gel part of a representative 5 % denaturing polyacrylamide gel separating ^{32}P-labeled alternative splice products from indicated developmental stages on top. Length of PCR products from splice variants are indicated at the right. # The 120 nt product can be either 3, 4c, 5 or 3, 3a, 4d, 5. M: Marker. **c, d** Analysis of alternative splicing from indicated developmental stages proximal (*Kpn*I) and distal (*Fok*I) of exon 4 from ^{32}P-labeled labeled forward (**c**) or return (**d**) primer after digestion with either *Kpn*I or *Fok*I on a representative 5 % denaturing polyacrylamide gels. ^ The 123 nt products are either 3, 4c, 4d, 5 or 4b, 4c, 4d, 5. ^ The 120 nt products are either 3, 3a, 4d, 5 or 4b, 4c, 5 or 3, 4c, 5. * The 78/80 nt products are either 4b, 5 or 3, 5 +/- 4d. M: Marker.

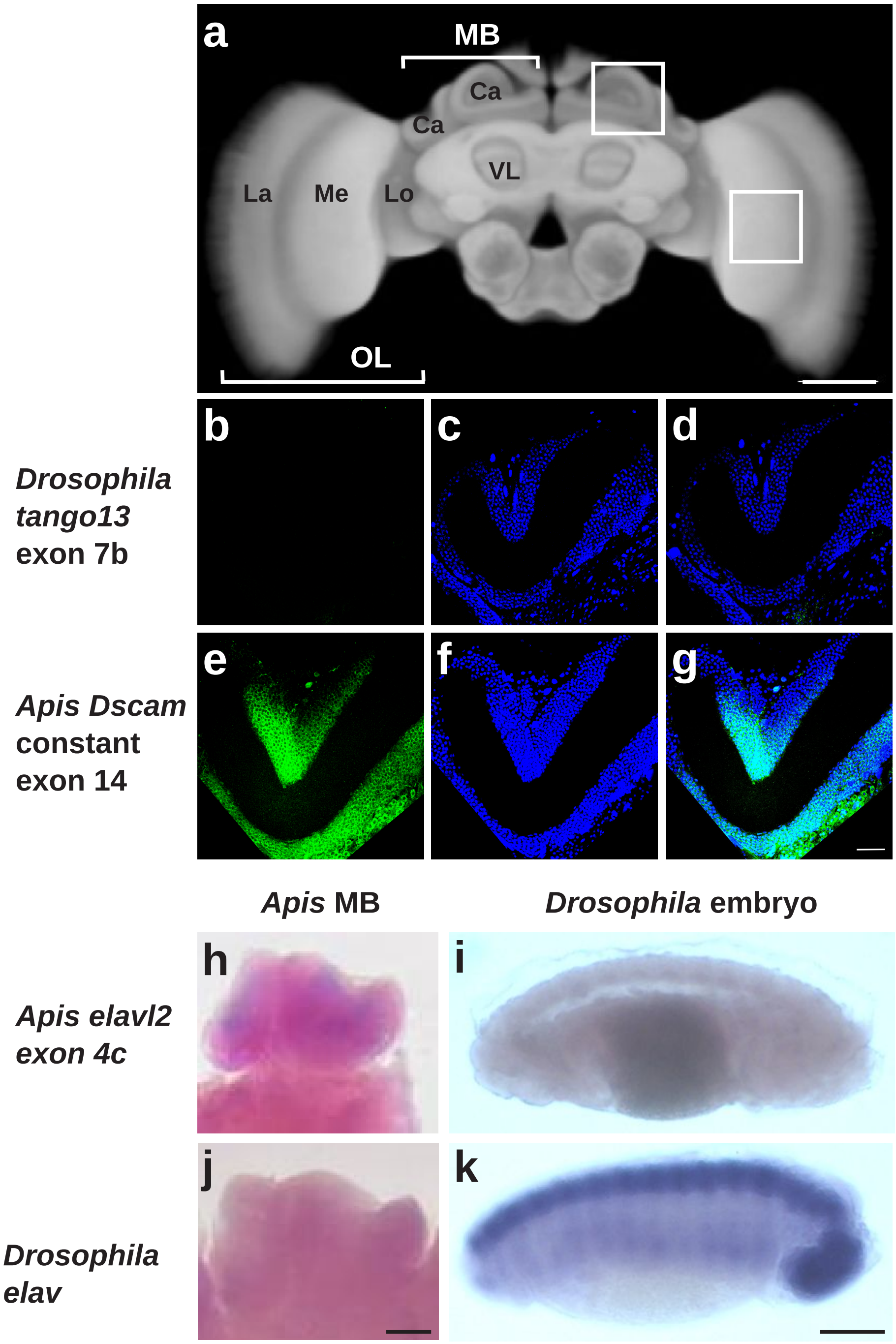

Supplementary Fig. 5 Representative control RNA in situ hybridizations in worker bees. a Frontal view of a honey bee brain showing the optic lobes (OL) and mushroom bodies (MB), two prominent paired structures. The optic lobes include the lamina (La), medulla (Me) and lobula (Lo), and the visible parts of the mushroom bodies are the calyces (Ca) and vertical lobes (VL). The square in the mushroom bodies indicates the area shown in panels b-g and in Figs 4a-f, j-o and 5a-i (mushroom body calyces). The square in the optic lobe shows the area shown in Fig 4g-i, p-r. The scale bar is 250 µm. **b-g** Control RNA in situ hybridization probes were against *Drosophila* alternatively spliced *tango13* exon 7b (**b**) and against ubiquitously expressed *Apis Dscam* constant exon 14 (**e**) in mushroom bodies visualized with FITC labeled secondary antibodies counterstained with DAPI to visualize nuclei (**c, f**) and merged pictures (**d, g**). Scales bar in I is 30 µm. **h-k** RNA in situ hybridization in bee mushroom bodies and *Drosophila* using a probe against bee elavl2 exon 4c (**h, i**) or a probe against *Drosophila elav* (**j, k**). Scale bars are 100 µm.

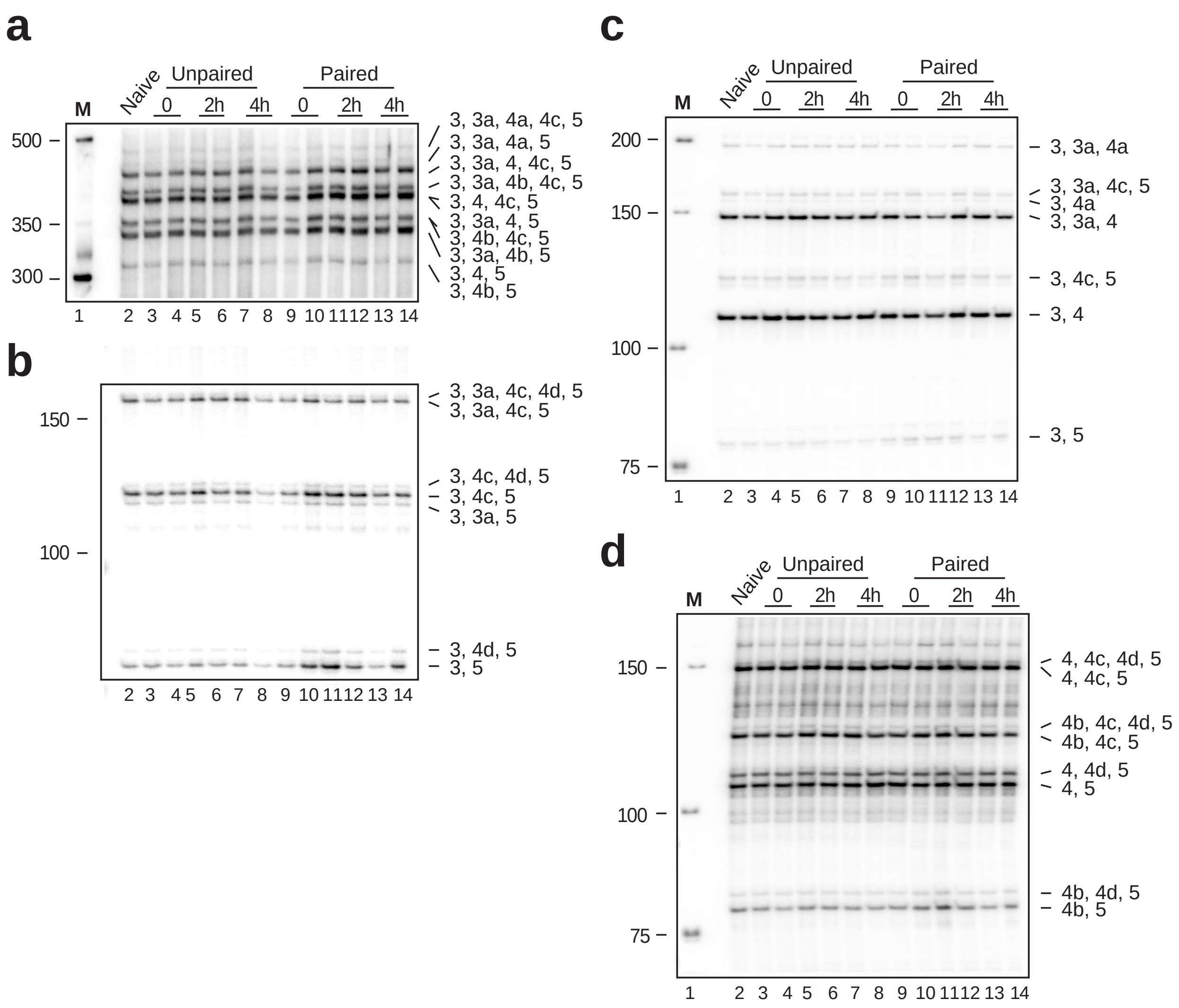

Supplementary Fig. 6 Representative gels of ELAVL2 alternative splicing after learning. a, b Top (**a**) and bottom (**b**) gel part of a representative 5 % denaturing polyacrylamide gel separating ^{32}P-labeled alternative splice products from naïve and trained bees from unpaired and paired conditioning of conditioned (sugar) and unconditioned (odor) stimuli at the indicated time after training. Length of PCR products from splice variants are indicated at the right. # The 120 nt product can be either 3, 4c, 5 or 3, 3a, 4d, 5. M: Marker. **c, d** Analysis of alternative splicing from naïve and trained bees from unpaired and paired conditioning of conditioned (sugar) and unconditioned (odor) stimuli at the indicated time after training proximal (*Kpn*I) and distal (*Fok*I) of exon 4 from ^{32}P-labeled labeled forward (**c**) or return (**d**) primer after digestion with either *Kpn*I or *Fok*I on a representative 5 % denaturing polyacrylamide gels. ^ The 123 nt products are either 3, 4c, 4d, 5 or 4b, 4c, 4d, 5. ^ The 120 nt products are either 3, 3a, 4d, 5 or 4b, 4c, 5 or 3, 4c, 5. * The 78/80 nt products are either 4b, 5 or 3, 5 +/- 4d. M: Marker.

Supplementary table 1: Oligonucleotides and RNA in situ probes

Oligonucleotides

AM elav F1
GCCGCCGGCGCGAACGGAATGGACACAGTCGTACAAC
AM elav R1 GCGTCTAGAGGCGCGCCTCTACGCCGCCTTGCTCTTGTTCGTCTTGAAGC
AM elav qF3
CCCTCTTCTCGAGCATTGGCGAGGTTG
AM elav qR3
GCCGTACGGGCTGAATAGATTCTCCAG
AM elav F2
GTCGCGGATACTTTGCGACAACATCAC
AM elav R2
CCCGGGTAGCATCGAGTTTGCCAATAGATC
AM ELAV T7 RNAi F1
GGAGCTAATACGACTCACTATAGGGAGAATGATGGCGAACGGAATGGACACAG
AM ELAV T7 RNAi R1
GGAGCTAATACGACTCACTATAGGGAGACTACGCCGCCTTGCTCTTGTTCGTCTTG
GFP T7 RNAi F1
GGAGCTAATACGACTCACTATAGGGAGACTGTTCACCGGGGTGGTGCCCATC
GFP T7 RNAi R1
GGAGCTAATACGACTCACTATAGGGAGACTTGTACAGCTCGTCCATGCCGAGAG
GST AM ELAV F1
GACGTGCCCGACTACGCAAGCCCCGGGATGGCGAACGGAATGGACACAGTC
GST AM ELAV R1
CGTCAGTCAGTCACGATGAATTGCGGCCGCTACGCCGCCTTGCTCTTGTTCGTC
RNA IS stem 1A
CTCAAACACATATATACATATACATATAGGGGTACATACATATATACATATATACTCGAG
RNA IS stem 1B
AGTATATATGTATATATGTATGTACCCCTATATGTATATGTATATATGTGTTTGAGGTAC
tango13A
TATGGTAAGCCAGATGCATGGGTGCAGGACAACACGTCGAAGTTAGATATCG
tango13B
AATTCGATATCTAACTTCGACGTGTTGTCCTGCACCCATGCATCTGGCTTACCATACTCG
AM elav 3a A
TCGAGACAACATCACCGTACGACAGTTTGTGACCGGCGGCGGAGACTATTTGCCCGGATT
GTCGAAAAGTACTGAATTCCTGCA
AM elav 3a B
GGAATTCAGTACTTTTCGACAATCCGGGCAAATAGTCTCCGCCGCCGGTCACAAACTGTC
GTACGGTGATGTTGTC
AM elav 4c A
TCGAGGCCGCTTCAGCACTGGCAAGGCCATGCTTGCCATTAACAAAGGCTTACAGAGGT
ACAGCCCGCAGTACTGAATTCCTGCA
AM elav 4c B
GGAATTCAGTACTGCGGGCTGTACCTCTGTAAGCCTTTGTTAATGGCAAGCATGGCCTTG
CCAGTGCTGAAGCGGCC

RNA in situ probes

Apis asELAV 3a
GGGAACAAAAGCTGGAGCTCCACCGCGGTGGCGGCCGCTCTAGAACTAGTGGATCCCCCGGGCTG
CAGGAATTCAGTACTTTTCGACAATCCGGGCAAATAGTCTCCGCCGCCGGTCACAAACTGTCGTACG
GTGATGTTGTCTCGAGTATATATGTATATATGTATGTACCCCTATATGTATATGTATATATGTGTTTGA
G

Apis asELAV 4c
GGGAACAAAAGCTGGAGCTCCACCGCGGTGGCGGCCGCTCTAGAACTAGTGGATCCCCCGGGCTG
CAGGAATTCAGTACTGCGGGCTGTACCTCTGTAAGCCTTTGTTAATGGCAAGCATGGCCTTGCCAGT
GCTGAAGCGGCCTCGAGTATATATGTATATATGTATGTACCCCTATATGTATATGTATATATGTGTTT
GAG

Apis asDscam 14
GGGAGAGTGTGCTGGTCGAGATCGTCGACGCGGATGGAGGTTGGGGGTCCGCTGGGCGCCTCCTC
GGCAGTGATTATGGTGACAGTGTCGGACGGGTCGGACGCGCCTATTTCGTTCTCGGCCACGATTCG
AGGTGATAAGTGGTGGCAGGCCTGAGATTGAACACTCCAGCCACGTTCTGTTGGGATCCAGGAACC
AGAACTCTGTCGATGTCGGTCTCCCACGAGCCTTTGCTTATCTTGTACTCGATCACGTAGCGCTTGAT
CGGGCTGTTCCCGTCGTACGGCGCCGCCCAGGAAAGTTGAACCGAGCGTCCAGACTTGTCCAACAC
CTTCAAACCGTACGGAACCTCGG

Drosophila asTango13 7b
GGGAACAAAAGCTGGAGCTCCACCGCGGTGGCGGCCGCTCTAGAACTAGTGGATCCCCCGGGCTG
CAGGAATTCGATATCTAACTTCGACGTGTTGTCCTGCACCCATGCATCTGGCTTACCATACTCGAGTA
TATATGTATATATGTATGTACCCCTATATGTATATGTATATATGTGTTTGAG
