# Supplementary material for: Dynamically expressed single ELAV/Hu orthologue elavl2 of bees is required for learning and memory
Source: Commun Biol. 2021 Oct 28;4:1234. doi: 10.1038/s42003-021-02763-1 (PMC8553928; doi:10.1038/s42003-021-02763-1)
Supplement: Supplementary file 4 — Description of Additional Supplementary Files [file 42003_2021_2763_MOESM4_ESM.pdf]

Description of Additional Supplementary Files

File name: Supplementary Data 1.
Description: Source data and uncropped gels/blots.
